# Seasonal bottom-up and top-down control of plankton in a hypereutrophic macrotidal lagoon on Brazil’s equatorial coast

**DOI:** 10.1007/s10661-026-15162-y

**Published:** 2026-03-16

**Authors:** Marco Valério Jansen Cutrim, Yago Bruno Silveira Nunes, Ana Karoline Duarte dos Santos Sá, Quedyane Silva da Cruz, Andrea Christina Gomes de Azevedo-Cutrim, Jordana Adorno Furtado

**Affiliations:** 1 Phycology Laboratory, Federal University of Maranhão (UFMA) - São Luís, Maranhão, Brazil; 2 Laboratory of Plant and Marine Biology, State University of Maranhão (UEMA) - São Luís, Maranhão, Brazil

**Keywords:** Community ordination, Cyanobacteria, Bioindicators, Grazing pressure, Nutrient enrichment

## Abstract

Tropical coastal lagoons often show pronounced seasonal forcing that modulates nutrient supply, light climate, and grazer pressure. We surveyed hypereutrophic Jansen Lagoon (São Luís Island, Brazil) in four campaigns during 2017 (rainy: March and April; dry: September and November) at nine near-surface stations sampled on ebb tide. Phytoplankton and zooplankton were collected and analyzed together with chlorophyll-a and nutrients to assess short-term community responses. Clustering and nMDS revealed clear rainy–dry segregation of communities, and dbRDA linked dry-season samples to higher salinity, turbidity, TP, and silicate, whereas rainy-season samples were associated with higher dissolved oxygen, Secchi depth, ammonium, and DIN. Generalized linear models explained 65% of phytoplankton variance: density increased with DIP and decreased with dissolved oxygen and with the rotifer *Filinia longiseta*, indicating concurrent bottom-up (nutrients, light/renewal) and top-down (grazing) controls. *Microcystis wesenbergii* and *M. aeruginosa* exhibited frequent peaks, underscoring eutrophic risk, though values remained below the bloom threshold applied here. Overall, bottom-up control predominated in the rainy season, whereas grazer pressure intensified in the dry season. Management should couple nutrient-load reductions with measures that shorten residence time, reduce resuspension, and restore macrophytes, with priority to urban margins and semi-enclosed embayments; routine tracking of DO percent saturation, DIN/DIP, Secchi depth, and chlorophyll-a is recommended for long-term assessment.

## Introduction

Coastal lagoons are shallow water bodies separated from the sea by natural or artificial barriers. Their physicochemical conditions, especially salinity, shift quickly over space and time under seasonal forcing and human influence (Cruz et al., 2018; Sadat-Noori et al., 2016). As interfaces between land and ocean, they support nutrient cycling, carbon processing, and biodiversity (Pérez-Ruzafa et al., 2019). Changes in salinity, oxygen, nutrients, and light ripple through food webs, altering community structure and ecosystem function (Gamito et al., 2019; Paturej et al., 2017).

In tropical cities, lagoons face mounting pressure from population growth and insufficient wastewater treatment. Nutrient enrichment drives eutrophication, the accumulation of nitrogen and phosphorus, harmful algal proliferation, and hypoxia (Béjaoui et al., 2018; Domingues et al., 2017; Paerl, 2009). Plankton respond quickly to these shifts and are widely used to diagnose contamination and trophic state (Casé et al., 2008; Gamito et al., 2019).

Zooplankton, the main grazers on algae, are sensitive to food quality and quantity, which shape their composition, growth, and reproduction (Sipaúba-Tavares & Bachion, 2002). In eutrophic waters, cyanobacteria often dominate and can suppress grazing through filamentous forms (Fulton & Paerl, 1987), toxicity (Fulton & Paerl, 1988), and poor nutritional value (Von Elert & Wolffrom, 2001). Filaments may even clog filtering structures (DeMott et al., 2001), tipping competition toward cyanobacteria and away from edible algae (Gragnani et al., 1999). Understanding this zooplankton–phytoplankton interplay is central to understanding how eutrophication unfolds.

In Brazil, most lagoon studies still focus on subtropical, heavily impacted systems, e.g., Lagoa Rodrigo de Freitas (Rio de Janeiro), where eutrophication has reshaped zooplankton (Souza et al., 2011). Work in tropical lagoons along the equatorial margin remains scarce. The Mundaú–Manguaba complex has revealed how nutrients and salinity structure zooplankton (Luz et al., 2022). Farther north, fewer lagoons and fewer studies exist; at Jansen Lagoon (Maranhão), recent research already points to strong urban and eutrophication signals (Cutrim et al., 2019). Worldwide, findings from eutrophic lakes support the value of zooplankton as sensitive indicators of water quality (García-Chicote et al., 2019; Muñoz-Colmenares et al., 2021).

Here, we examine spatial and seasonal plankton dynamics in an urban, hypereutrophic tropical lagoon on Brazil’s equatorial coast. We (i) describe zooplankton and phytoplankton across seasons and stations; (ii) test relationships between nutrients, salinity, oxygen, light, and phytoplankton (bottom-up control); and (iii) evaluate whether grazer groups exert top-down effects. This study offers a comprehensive assessment of plankton structure and dynamics in a tropical eutrophic lagoon strongly influenced by tidal exchange, high turbidity, and urban pressure. The results contribute to a broader understanding of how hydrodynamic variability and nutrient enrichment shape plankton communities in tropical coastal systems and provide valuable guidance for monitoring and managing similar urbanized lagoons and estuaries.

## Materials and methods

### Study area and sampling stations

**Fig. 1:**
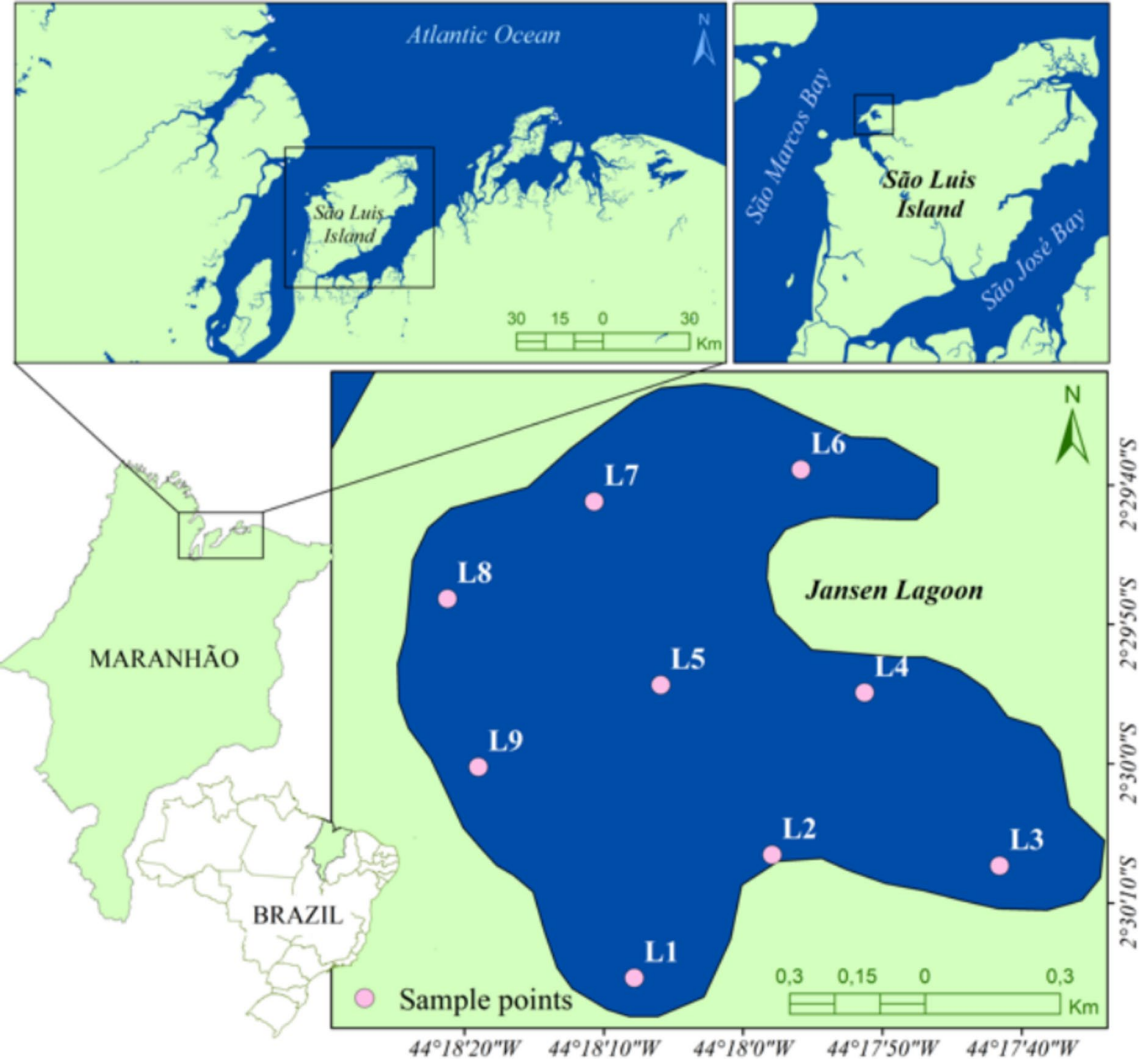
Location of the study area with sampling points (L1–L9), Jansen Lagoon, São Luís Island–Maranhão, Brazil

Jansen Lagoon is a coastal lagoon formed by damming Ana Jansen Creek. It spans ~ 140 ha, averages ~ 1.5 m in depth, and is bordered east by mangrove forest (Cutrim et al., 2019). The lagoon is in the northwestern sector of São Luís Island, northern Brazil, near 02°29′08″ S; 044°18′02″ W (Fig. 1). It connects to São Marcos Bay via the Ana Jansen stream and is influenced by semidiurnal macrotides that can exceed 4 m (Dados Maregráficos e Fluviométricos | CHM).

To represent the principal environmental settings, we established nine stations (L1–L9) along gradients of marine influence, urban inputs, hydrodynamic retention, and macrophyte cover (WGS84, DMS). L1 (02°30′03′′ S 44°18′18′′ W) is in the southwest area of the lagoon, which is influenced by a supply of salt water from the São Marcos Bay. Southern estuarine station L9 (02°30′03″ S; 44°18′18″ W) marks the transition to São Marcos Bay. L2 (02°30′16″ S; 44°18′21″ W), L3 (02°30′23″ S; 44°18′13″ W), and L7 (02°30′08″ S; 44°18′11″ W) lie on urban shores receiving domestic sewage, where the organic load and turbidity are typically relatively high. L4 (02°30′19″ S; 44°18′04″ W) and L6 (02°30′23″ S; 44°18′39″ W) are semi-enclosed embayments with reduced water exchange and degraded mangrove stands (*Rhizophora mangle* Linnaeus, *Avicennia germinans* (Linnaeus) Linnaeus) prone to local stagnation. L5 (02°30′06″ S; 44°18′32″ W) represents the central body of the lagoon near a mangrove islet, and L8 (02°30′23″ S; 44°18′32″ W) lies in a reach dominated by submersed macrophytes (*Ruppia maritima *Linnaeus).

#### Sampling design

Sampling was conducted at nine stations during ebb tide in four field campaigns: two during the rainy season (March and April 2017) and two during the dry season (September and November 2017) to ensure consistent spatial and seasonal coverage across the study area. All plankton and environmental measurements were taken from the surface layer (~ 0.5 m) to ensure consistency. To standardize tidal influence, we sampled within an ~ 2 h window around mid-ebb, scheduling each campaign with DHN/CHM (Diretoria de Hidrografia e Navegação/Dados Maregráficos e Fluviométricos | CHM) tide tables and noting the local time and tidal stage at every station, and the order of stations was kept constant across campaigns to limit temporal drift.

#### Zooplankton sampling and analysis

Zooplankton were collected with horizontal subsurface tows (~0.5 m depth) via a 120 µm plankton net coupled to a General Oceanics® flowmeter. At each station, we performed two replicate tows, each lasting 3–5 min (adjusted to local hydrodynamics and debris). The samples were immediately preserved in buffered formaldehyde (4% final). We used a flowmeter to compute the filtered volume (m^3^) and standardized the abundances to ind. m⁻^3^. If a tow was compromised (air entrainment, net clogging, or flowmeter failure), we discarded it and repeated the process.

The choice of a 120 µm mesh was justified by the eutrophic–hypereutrophic and highly turbid conditions of the São Marcos Estuarine Complex, where suspended solids and dense filamentous or coccoid algae, particularly during ebb tides, can rapidly clog finer meshes. Using a smaller mesh (e.g., 64 µm) would have severely reduced the effective filtered volume and impaired comparability across stations and tidal phases. Thus, the 120 µm mesh represented an optimal compromise, ensuring reproducible sampling efficiency while targeting the mesozooplankton fraction (copepodites and adults), which are key components of top-down grazing in estuarine food webs. As a methodological trade-off, microzooplankton and small rotifers may be underrepresented; therefore, grazing inferences in this study refer conservatively to the mesozooplankton captured with this mesh size.

In the laboratory, we identified organisms to the lowest feasible taxonomic level under compound and stereomicroscopes. Rotifers and nauplii were counted in a Sedgwick–Rafter chamber (400×); copepodites and adults were counted in open chambers under a stereomicroscope. The taxonomic framework of copepods was based on the works of Björnberg (1981), Boltovskoy (1999), and Bradford-Grieve et al. (1999). Abundances were standardized to individuals per cubic meter (ind. m⁻^3^) based on the filtered volume recorded by the flowmeter.

#### Phytoplankton sampling and analysis

For phytoplankton, we collected 250 mL of subsurface water using a Van Dorn bottle and preserved samples with Lugol’s iodine (~ 1% v/v). Counts followed the Utermöhl (1958) method under ×400 magnification. Organisms were enumerated as they occurred in nature: single cells, colonies, filaments, or coenobia.

For colonial and filamentous taxa, we estimated the mean number of cells per individual unit by counting 25–30 representative colonies/filaments of each species. Phytoplankton density (cells L⁻^1^) was then derived by multiplying the abundance of individuals (ind. mL⁻^1^) by the mean number of cells per individual, following the procedures of Villafañe and Reid (1995). A minimum of 100 fields was counted per sample. Blooms were defined as ≥ 1 × 10⁶ cells L⁻^1^ for any given taxon (Livingston, 2007).

Phytoplankton taxa were identified using Bicudo and Menezes (2006) and Round et al. (2007), with nomenclature checked against the currently accepted names in Guiry and Guiry (2025).

#### Environmental variables, nutrients, and chlorophyll-a

The in situ measurements included temperature, salinity, and pH at ~ 0.5 m using a multiparameter probe (HI-9828, Hanna®; calibrated daily), turbidity (NTU) measured with a portable turbidimeter (model 2020), and Secchi depth (20 cm white disk) recorded before any disturbance to the water column.

For nutrients, we collected 2 L of surface water (~ 0.5 m), stored the samples on ice, and processed them as soon as possible (typically within 6–8 h). We computed DIN as NH₄⁺-N + NO₂⁻-N + NO₃⁻-N and DIP as PO₄^3^⁻-P; we also measured TP and dissolved silicate (SiO₂-Si). The methods followed APHA (2012) and classical colorimetry (Grasshoff et al., 1983; Koroleff, 1983; Strickland & Parsons, 1972), with results in µmol L⁻^1^.

For chlorophyll-a (Chl-a), we analyzed two technical replicates per station: 250 mL was filtered on GF/F (0.7 µm), extracted in 90% acetone under low light, and read on a UV–Vis spectrophotometer (Thermo Scientific Evolution™ 201). The concentrations (µg L⁻^1^) were determined via the Parsons–Strickland equations, as described by Strickland and Parsons (1972). Each batch included procedural blanks.

#### Exclusion criteria and QA/QC

Quality assurance (QA) and quality control (QC) refer to the procedures adopted to ensure the reliability and accuracy of field and laboratory measurements.

We excluded samples when (i) the flowmeter malfunctioned or the tow was aborted/clogged, (ii) field metadata were incomplete, or (iii) laboratory QC failed (e.g., replicate divergence beyond acceptance limits). All exclusions and reasons were documented.

Before every campaign, the probe underwent calibration and was subsequently checked for drift. Readings outside the acceptable range were taken again. For nutrients, each campaign included field duplicates and reagent blanks; fresh 5-point calibration curves (*R*^2^ ≥ 0.995) were prepared, with detection limits derived from low-level standards and blanks; and spikes or certified materials confirmed acceptable recoveries. For Chl-a, we compared replicates, handled extracts cold and dark, and applied an acidification step to correct for pheophytin; outliers were re-extracted or flagged.

#### Diversity indices and statistical analyses

We computed species richness (Margalef), diversity (Shannon–Wiener), and evenness (Pielou) from relative abundances in PAST (Paleontological Statistics) v3. Indices were calculated per sample after standardization (zooplankton: ind. m⁻^3^; phytoplankton: cells L⁻^1^).

Before hypothesis testing, we assessed distributions with the Shapiro–Wilk (normality) and Levene tests (homogeneity). When assumptions were not met, we applied log₁₀ transformation. Two-way ANOVAs were then performed to test the effects of season (rainy and dry) and sampling station (L1–L9) on environmental and biological variables. When normality or homoscedasticity assumptions were violated, Kruskal–Wallis tests were applied as non-parametric alternatives, with *α* = 0.05.

To identify seasonal indicators, we applied the indicator value (IndVal) (Dufrêne & Legendre, 1997) to zooplankton abundances grouped by season (rainy vs. dry), considering that *p* < 0.05 was significant (permutation tests).

To explore multivariate structure, we conducted non-metric multidimensional scaling (nMDS) and hierarchical cluster analysis on square-root transformed phytoplankton and zooplankton abundance matrices using Bray–Curtis similarities. nMDS solutions were optimized with 999 iterations and stress minimization, while clusters were generated using group-average (UPGMA) linkage. The nMDS provided an ordination of seasonal dissimilarities, whereas the cluster dendrogram confirmed the grouping patterns.

Relationships among community structure and environmental drivers were examined using distance-based redundancy analysis (dbRDA). Community data were square-root transformed, environmental variables were centered and scaled, and model significance was evaluated via permutation tests (*n* = 999).

Given the selectivity of the zooplankton gear (120-µm mesh focusing on mesozooplankton), predictors derived from zooplankton were interpreted as mesozooplankton proxies. To assess robustness to collinearity and size-selective sampling, we additionally fit a VIF-filtered GLM (retaining predictors with a VIF ≤ 10) to log₁₀-transformed phytoplankton density (Gaussian, identity link).

## Results

### Physical, chemical, and biological variables of Jansen Lagoon

Water temperature ranged from 25.5 to 26.6 °C and differed significantly between seasons (two-way ANOVA, *F* = 4.26, *p* < 0.001) but did not differ among stations. Salinity was markedly greater in the dry season than in the rainy season and varied significantly between seasons (ANOVA, *F* = 13.8, *p* < 0.001). Dissolved oxygen (DO) also varied seasonally (ANOVA, *F* = 13.01, *p* < 0.0001), with the lowest values occurring in the dry months. Nutrients displayed clear seasonality: DIN was dominated by NH₄⁺ (~ 85%), whereas DIP was higher in the dry season. Community indices were consistently larger in the rainy season; zooplankton showed greater diversity and richness with high evenness, and phytoplankton followed the same pattern. All seasonal contrasts were significant (Table 1).

**Table 1:** Seasonal means (± SDs) of physicochemical and biological variables in Jansen Lagoon (rainy vs. dry; 2017; surface ≈0.5 m; ebb tide). *F* gives the test statistic from two-way ANOVA; where assumptions were not met, the Kruskal–Wallis result is reported for seasonal contrast (see “Methods”). Asterisks denote significant seasonal differences (*p < 0.05). Abbreviations: *DO* dissolved oxygen, *DIN* dissolved inorganic nitrogen (NH₄⁺ + NO₂⁻ + NO₃⁻), *DIP* dissolved inorganic phosphorus (PO₄³⁻–P), *TP* total phosphorus, *SiO₂* dissolved silicate, *Chl-a* chlorophyll-a

Variables	Rainy	Dry	*F*	*p*
Depth (m)	0.9 ± 0.3	0.7 ± 0.4	2.8	< 0.001*
Secchi (m)	0.8 ± 0.2	0.4 ± 0.2	41.5	< 0.001*
Temperature (°C)	25.5 ± 0.9	26.6 ± 0.5	4.3	< 0.001*
pH	8.7 ± 0.3	8.9 ± 0.1	6.6	< 0.001*
Salinity	6.1 ± 1.5	31.8 ± 2.5	13.8	< 0.001*
Turbidity (NTU)	9.0 ± 2.5	34.0 ± 22.4	23.5	0.011
DO (mg L^−1^)	2.9 ± 1.3	1.1 ± 0.2	13.0	< 0.001*
NO^−^_2_ (µmol L^−1^)	0.1 ± 0.1	0.2 ± 0.1	7.6	< 0.001*
NH^+^_4_ (µmol L^−1^)	43.9 ± 72.3	9.4 ± 2.8	6.8	< 0.001*
NO^−^_3_ (µmol L^−1^)	2.9 ± 2.5	6.0 ± 6.4	4.7	0.035
DIN (µmol L^−1^)	44.0 ± 72.3	15.6 ± 7.2	7.9	0.006
DIP (µmol L^−1^)	0.3 ± 0.3	0.5 ± 0.2	6.6	< 0.001*
SiO_2_ (µmol L^−1^)	1.0 ± 0.3	4.0 ± 0.7	16.8	< 0.001*
TP (µmol L^−1^)	4.3 ± 2.4	3.9 ± 1.0	24.8	< 0.001*
Chl-a (µg L^−1^)	80.2 ± 61.6	246.3 ± 40.7	52.0	< 0.001*
Phytoplankton—Shannon diversity index	0.96 ± 0.5	1.03 ± 0.2	11.1	< 0.001*
Phytoplankton—Richness (S)	1.5 ± 0.2	0.92 ± 0.2	3.1	< 0.001*
Phytoplankton—Evenness (Pielou’s J)	0.3 ± 0.2	0.38 ± 0.1	83.2	< 0.001*
Zooplankton—Shannon diversity index	0.9 ± 0.1	0.85 ± 0.1	114.9	< 0.001*
Zooplankton—Richness (S)	10.2 ± 7.7	2.12 ± 0.3	98.4	< 0.001*
Zooplankton—Evenness (Pielou’s J)	0.83 ± 0.8	0.83 ± 0.1	27.5	< 0.001*

### Planktonic community composition

The zooplankton community comprised 25 taxa, dominated by rotifers (57.0% of taxa) and copepods (23.8%), with protozoans and tintinnids contributing 9.5% each. During the rainy season, rotifers and tintinnids occurred at all stations (100% occurrence). In the dry season, tintinnids were absent, and several rotifer and copepod taxa also dropped out of the assemblage (Table 2). IndVal analysis identified a suite of indicator taxa spanning both seasons (e.g., *Brachionus plicatilis*, *B. angularis*, *Attheyella fuhrmanni*, Harpacticoida, *Filinia longiseta*, *Hexarthra mira*, *Lepadella* sp., and *Parvocalanus crassirostris*), as detailed in Table 2.

**Table 2:** Abundance, occurrence total, and IndVal values for zooplankton taxa in Jansen Lagoon

	Rainy		Dry		IndVal	*p*
	ABU (ind m^−3^)	TO (%)	ABU (ind m^−3^)	TO (%)		
**Tintinidae**						
* Favella ehrenbergi* (Claparède & Lachmann, 1858) Jörgensen, 1924	0.07	0.05	-	-	11.11	0.0001
* Tintinnopsis compressa* Daday, 1887	0.03	0.05	-	-	11.11	0.0001
**Rotifera**						
* Asplanchna sieboldii* (Leydig, 1854)	1.10	4.25	38.89	0.96	100	0.4229
Brachionus angularis* Gosse, 1851	3.73	4.89	339.61	6.27	100	0.0401
* Brachionus calyciflorus* Pallas, 1766	1.84	2.39	13.89	0.12	66.66	0.2381
Brachionus plicatilis* (Müller, 1786**)**	13.19	17.10	1583.53	26.17	100	0.019
* Brachionus urceolaris* (Müller, 1766)	4.56	4.41	426.39	6.63	100	0.0790
* Colurella uncinate deflexa* (Ehrenberg, 1834)	2.11	2.87	107.99	1.48	100	0.3651
* Colurella* sp.*	0.15	0.42	-	-	33.33	0.0058
* Euchlanis dilatata* f. *lucksiana* (Ehrenberg, 1830)	4.81	4.35	568.75	6.83	100	0.3033
Filinia longiseta* (Ehrenberg, 1834)	3.78	3.98	0.74	0.08	100	0.0279
* Hexarthra mira* (Hudson, 1871)	9.95	13.12	-	-	88.88	0.0982
* Leocane lunaris* Ehrenberg, 1832	0.15	4.20	15.63	0.60	88.88	0.0777
* Lepadella* sp.	0.19	2.81	-	-	77.77	0.3843
**Copepoda**						
Apocyclops panamensis* (Marsh, 1913)	0.65	4.35	379.54	7.27	100	0.0008
* Acanthocyclops* sp	0.12	2.07	-	-	100	0.2356
Atheylla fuhrmanni* (Thiébaud, 1912)	0.49	6.80	696.40	13.03	100	0.0004
* Parvocalanus crassirostris* Dahl F., 1894	0.79	3.08	-	-	100	0.0670
Harpacticoida *	1.18	7.59	460.94	9.95	100	0.0038
Copepodites/nauplii	11.72	9.77	894.79	14.27	100	0.8443
**Protozoa**						
* Difflugia* sp.	-	0.53	125.05	2.44	100	0.2245
* Arcella* sp.	-	-	95.16	2.04	100	0.0716
**Others**						
Nematoda	0.02	0.37	87.50	1.08	77.77	0.1224
Polychaeta larvae*	0.07	0.37	29.94	0.44	55.55	0.0036
Polychaeta (Phyllodocidae)	-	0.16	23.26	0.32	55.55	0.2167
**TOTAL**	**60.70**	**100.00**	**6915.89**	**100.00**	**-**	**-**

*ABU* abundance, *TO* total occurrence

*Indicators species according to Indval values (IndVal > 25%)

The phytoplankton community included 74 taxa distributed among Cyanophyta (47.30%), Bacillariophyta (37.84%), Chlorophyta (8.11%), Euglenophyta (1.52%), and Dinophyta (1.35%). The highest densities occurred in the rainy season at L6 (48.660 × 10^3^ cells L⁻^1^) and L5 (42.060 × 10^3^ cells L⁻^1^), and phytoplankton density varied significantly across seasons and among stations (*p* < 0.05). *Microcystis* spp. dominated Cyanobacteria; *Stephanocyclus meneghinianus* (Kützing) Kulikovskiy, Genkal & Kociolek peaked in the rainy season at L1 (2.56 × 10^3^ cells L⁻^1^) and L6 (1.05 × 10^3^ cells L⁻^1^). *M. wesenbergii* (Komárek) Komárek ex Komárek reached its highest densities during the dry season at almost all stations except L9, ranging from 1.40 × 10^3^ cells L⁻^1^ (L3) to 23.34 × 10^3^ cells L⁻^1^ (L7) in the rainy season. *M. aeruginosa* (Kützing) Kützing also peaked at most stations in the dry season (absent at L9), ranging from 1.30 × 10^3^ cells L⁻^1^ (L5) to 18.05 × 10^3^ cells L⁻^1^ (L4), and reached relatively high values in the rainy season (up to 46.16 × 10^3^ cells L⁻^1^ at L6, with a minimum of 9.00 × 10^3^ cells L⁻^1^ at L7).

### Factors affecting the planktonic community

Modeling phytoplankton density as the response variable, the stepwise-backward GLM explained 65% of the variance (adjusted *R*^2^ = 0.65; Table 3). The final multivariate model revealed an integrated relationship in which higher phytoplankton density occurred under elevated dissolved inorganic phosphorus (DIP) and lower dissolved oxygen, combined with reduced abundance of *F. longiseta*. Together, these predictors represented the composite structure captured by the model: phytoplankton density =  − 0.94·DO − 0.36·*F. longiseta* + 0.46·DIP. Although *B. plicatilis* had a negative coefficient, its very high variance inflation factor (VIF ≈ 129) indicated severe collinearity, precluding meaningful interpretation. No other predictors were statistically significant.

**Table 3:** Generalized linear model (GLM) for phytoplankton density in Jansen Lagoon via stepwise backward selection. Reported are standardized coefficients (*β*), standard error (SE), *t* = β/SE, (*T*), *p* values, the variance inflation factor (VIF), and significance. The final multivariate model (adjusted *R*^2^ = 0.65) identified dissolved oxygen (DO), DIP (PO₄^3^⁻–P), *Brachionus plicatilis* and *Filinia longiseta* as significant predictors (*α* = 0.05). Predictors with a VIF > 10 are flagged for collinearity

Predictor	*β* (std)	SE(*β*)	*T* = *β*	*p* value	VIF	Signif	Collinearity flag (VIF > 10)
Dissolved oxygen (DO)	−0.94	0.17	−5.48	0.0001	2.21	***	No
Salinity	−0.07	0.26	−0.26	0.7990	4.93		No
DIP (PO_4_^3−^-P)	0.46	0.17	2.86	0.0119	1.91	*	No
*Brachionus angularis*	0.41	0.65	0.63	0.5387	31.15		Yes
*Brachionus plicatilis*	−3.58	1.32	−2.71	0.0161	129.51	*	Yes
*Filinia longiseta*	−0.36	0.13	−2.70	0.0166	1.30	*	No
Polychaeta larvae	0.82	0.45	1.84	0.0851	14.81		Yes
*Colurella* sp.	−0.15	0.3	−1.22	0.2376	1.16		No
Harpacticoida	−1.09	0.55	−1.85	0.0837	22.94		Yes
*Attheyella fuhrmanni*	0.65	0.54	1.21	0.2433	21.41		Yes
Total zooplankton abundance	2.13	1.36	1.57	0.1374	137.42		Yes

Significance: ***p < 0.001, *p < 0.05

For *Synechococcus* sp., the GLM accounted for 69% of the variance (adjusted *R*^2^ = 0.69) and identified a combined pattern linking higher *Synechococcus* abundance to conditions of increased dissolved oxygen and greater abundance of *A. fuhrmanni* and polychaete larvae. These predictors jointly characterized the ecological setting associated with *Synechococcus* proliferation, rather than functioning as isolated explanatory factors.

For *C. turgidus*, the best-fitting model (adjusted *R*^2^ = 0.75) indicated an integrated response shaped by the combined influence of *Colurella* sp., dissolved oxygen, salinity, and chlorophyll-a. Among these predictors, only chlorophyll-a exhibited statistical support (*p* < 0.05), contributing to a negative association with *C. turgidus* density. The remaining predictors contributed to the multivariate structure of the model but did not reach statistical significance.

### Seasonal structure and indicator patterns

**Fig. 2:**
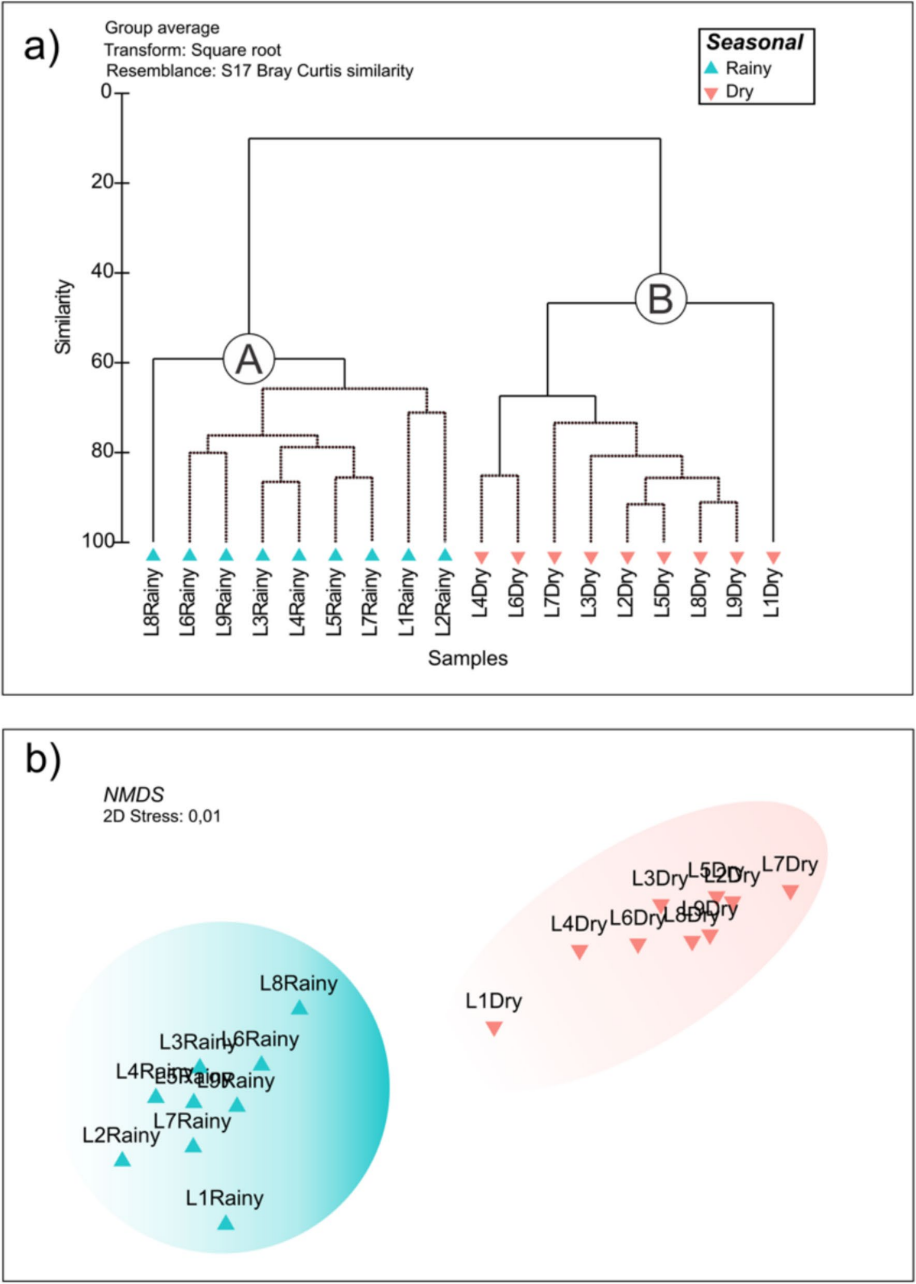
Seasonal structure of the planktonic community. **a** Cluster analysis (Bray–Curtis, square-root, UPGMA) resolves two groups—GI (rainy) and GII (dry); the red dashed line marks the ~12.86% cutoff. **b** nMDS of the same data (stress = 0.01) with the rainy season (green triangles) and dry season (blue inverted triangles); shaded hulls outline the two clusters

Multivariate analyses were performed separately for phytoplankton and zooplankton abundance matrices (square-root transformed, Bray–Curtis similarity) to identify seasonal community patterns without mixing datasets of different units. Hierarchical clustering (Bray–Curtis on square-root-transformed data, group-average linkage) and nMDS both revealed a clear seasonal separation of communities (Fig. 2a, b). The dendrogram (Fig. 2a) resolved two coherent clusters, with all rainy-season samples in GI and all dry-season samples in GII. The nMDS (Fig. 2b; stress = 0.01) corroborated this split, with rainy samples forming a tight cluster on the left and dry samples forming a compact cluster on the right. Notably, L1Dry plotted at the edge of the dry cluster, and L8Rainy lay farthest from the rainy centroid, indicating modest within-season heterogeneity. This multivariate pattern was consistent with the univariate contrasts and with the environmental gradients highlighted by the dbRDA.

**Fig. 3:**
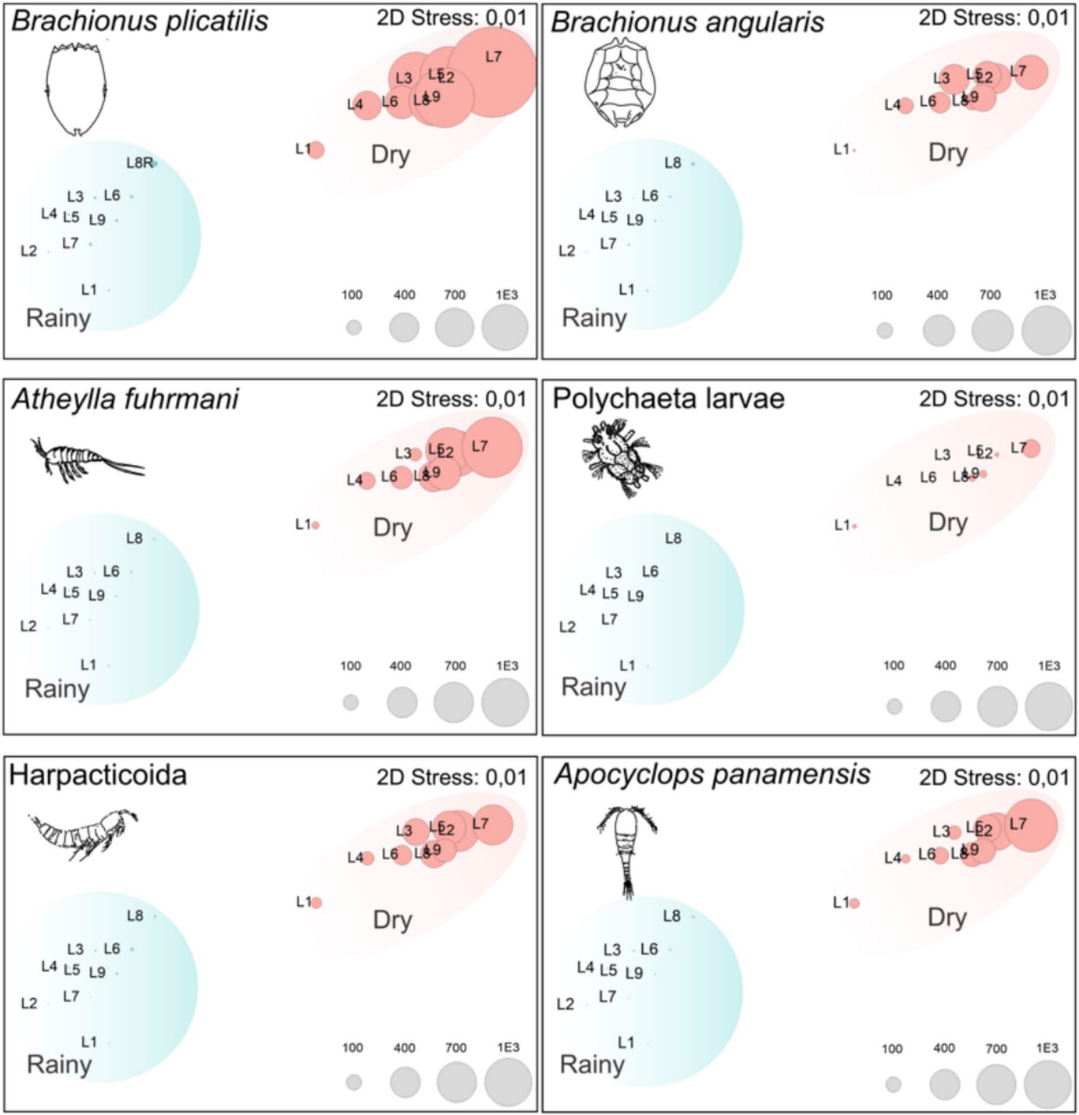
Taxon-specific abundance overlays the nMDS. nMDS ordination via Bray–Curtis on square-root-transformed data (2D stress = 0.01). Each panel shows one taxon (*B. plicatilis*, *B. angularis*, *A. fuhrmanni*, Polychaeta larvae, Harpacticoida, *A. panamensis*, *Colurella* sp., *F. longiseta*) as bubbles plotted at the sample scores; bubble diameter scales with abundance (ind. m⁻^3^) with panel-specific scales shown at the bottom-right

Indicator patterns mirrored this seasonal partitioning. Dry-season dominance was observed for *B. plicatilis*, *B. angularis*, *A. fuhrmanni*, Polychaeta larvae, Harpacticoida, and *A. panamensis*, with a marked peak at L7. In contrast, *F. longiseta*, *H. mira*, *Lepadella* sp., and *P. crassirostris* were more common and achieved higher IndVal during the rainy season (Fig. 3).

The bubble overlays on the nMDS (stress = 0.01; panel-specific abundance scales) reinforced the seasonal split seen in the dendrogram. The dry-season samples contained the largest bubbles for *B. plicatilis*, *B. angularis*, *A. fuhrmanni*, Polychaeta larvae, Harpacticoida, and *C. panamensis*, with a clear maximum at L7Dry and secondary peaks around L3–L5Dry. In contrast, *F. longiseta* was primarily associated with the rainy-season cluster, most notably at L8Rainy with a single high value at L1Dry; *Colurella* sp. was consistently rare in both seasons. Overall, taxa with higher abundances in the dry cluster tracked the conditions identified for the dry period (higher salinity and DIP/SiO₂), whereas those concentrated in the rainy cluster aligned with higher DO and DIN, which was consistent with the dbRDA patterns (Fig. 3).

The dbRDA captured 38.68% of the adjusted variation, with axis 1 = 26.54% and axis 2 = 12.14% (Fig. 3). The dry-season side of the gradient was associated with relatively high salinity, temperature, pH, turbidity, TP, and silicate contents, grouping taxa such as *B. plicatilis*, *B. angularis*, *B. urceolaris*, *E. dilatata* f. *lucksiana*, *Difflugia* sp., Protozoa sp., and Phyllodocidae. Conversely, the rainy-season side was associated with greater DO, Secchi depth, ammonium, and DIN concentrations, and *Lepadella* sp., *F. longiseta*, and *P. crassirostris* were associated with these conditions.

### Top-down and bottom-up controls on plankton

Across stations and seasons, phytoplankton dynamics reflected the joint influence of bottom-up and top-down factors. In the GLM, dissolved inorganic phosphorus (DIP) was a positive predictor of phytoplankton density (*β* = 0.46, *p* = 0.012), while the abundance of *F. longiseta* showed a negative effect (*β* =  − 0.36, *p* = 0.017), indicating concurrent effects of nutrient availability and grazing pressure. The rainy-season side of the ordinations aligned with higher DIN and greater light penetration, whereas the dry-season cluster was associated with higher abundances of grazing taxa, including *B. plicatilis*, *B. angularis*, *A. fuhrmanni*, Harpacticoida, and Polychaeta larvae (Fig. 3). The spatial and seasonal overlap between higher grazing taxa and elevated DIP concentrations in the dry period was consistent with the patterns captured by the dbRDA and the univariate contrasts (Table 1).

## Discussion

### Seasonal context in a hypereutrophic lagoon

Seasonality structured the system sharply. The dry-season waters presented higher salinity and DIP from evaporation and reduced freshwater input, whereas the rainy-season waters presented higher DO and DIN (NH₄⁺) and greater water renewal (Table 1). Despite the DIP peak in the dry period, the phytoplankton density and diversity were greater in the rainy season than in the dry period, which was consistent with the higher Secchi and lower salinity in the dry period (Fig. 2a, b). Similar seasonal forcings have been reported for tropical coastal lagoons (e.g., Branco et al., 2008; Delgadillo-Hinojosa et al., 2008; Maia-Barbosa et al., 2014). More broadly, coastal lagoons exhibit pronounced spatiotemporal gradients, especially in terms of salinity and nutrients, under strong anthropogenic pressure; these gradients are primary vectors shaping plankton distributions and indicator species selection (Hemraj et al., 2017), a rationale fully consistent with our results. In macrotidal settings, tidal pumping can further reorganize zooplankton structure across the tidal cycle, reinforcing the seasonal signal (Krumme & Liang, 2004).

### Bottom-up control: nutrients, light, and flushing

Two lines of evidence point to bottom-up forcing. First, the GLM identified DIP as a positive predictor of phytoplankton (*β* = 0.46, *p* = 0.012; Table 3). Second, the rainy-season samples plotted toward higher DIN and greater water transparency (higher Secchi depth) in the ordinations (Fig. 2b). Together, these results explain why phytoplankton increased in the rainy months even with lower DIP concentrations: light availability, nitrogen supply, and hydrodynamic renewal (flushing) appear to promote phytoplankton growth under better-oxygenated conditions. In this study, *flushing* refers to tidally driven water renewal inferred from the strong semidiurnal tides and high current velocities that enhance dilution and export processes during the rainy season. Although Secchi depth and phytoplankton density were not directly correlated in a bivariate sense, their association in the ordination patterns reflects the combined influence of improved light climate and hydrodynamic exchange, consistent with lagoon studies using plankton as bioindicators (Hemraj et al., 2017).

Although tropical Brazil does not experience a classical monsoon, the ITCZ-driven rainy season delivers comparable freshwater and nutrient pulses that reorganize salinity, turbidity, and residence time. In a monsoon-forced tropical bay (Marudu Bay, Malaysia), increases in nitrate triggered centric-diatom blooms (*Chaetoceros*, *Bacteriastrum*), whereas high ammonium suppressed them; high silica favored pennate diatoms, and silica depletion rapidly terminated blooms; additionally, high zooplankton abundance limited bloom development toward the end of the wet phase. These mechanisms provide a useful analogue for our system, helping to explain why rainy-season nutrient inputs and flushing structure phytoplankton composition and bloom dynamics here (Tan & Ransangan, 2017).

### Top-down control: grazer pressure and consumer structure

Evidence for top-down regulation has also emerged. The GLM revealed negative partial effects of *F. longiseta* (*β* =  − 0.36, *p* = 0.017) and *B. plicatilis* (*β* =  − 3.58, *p* = 0.016; high collinearity noted) on phytoplankton (Table 3), which was consistent with rotifer grazing. The nMDS results highlighted the dry-season dominance of grazing taxa, *B. plicatilis*, *B. angularis*, *A. fuhrmanni*, Harpacticoida, and Polychaeta larvae, with a clear maximum at L7 (Fig. 3). This consumer–producer coupling aligns with evidence that zooplankton can modulate phytoplankton, water transparency, and nutrients (Bess et al., 2021). Ecologically, *B. plicatilis* is well known for its tolerance to eutrophic conditions and broad salinity range (Arcifa et al., 1994; Castilho Noll et al., 2023; Sládeček, 1983), supporting its ecological success here.

Collectively, these results indicate that top-down pressure was exerted primarily by the mesozooplankton captured with the 120-µm net. Because this mesh size undersampled microzooplankton, particularly small rotifers, the magnitude of consumer effects reported here should be interpreted with caution, and further studies using finer meshes are needed to better resolve these interactions.

Seasonal dilution experiments in the monsoon-driven Chilika Lagoon (India) demonstrate that microzooplankton frequently remove a large share of phytoplankton standing stock and production (often g/k > 1). These findings indicate strong top-down regulation in eutrophic–mesohaline waters (Singh et al., 2025).

### Cyanobacteria, oxygen stress, and feedback

Frequent peaks of *M. wesenbergii* and *M. aeruginosa* fit the lagoon’s eutrophic status (Imai et al., 2009; Li & Xiao, 2016). Although counts remained below the bloom threshold defined in Methods (≥ 10⁶ cells L⁻^1^), such density peaks can depress DO through respiration and decay, reinforcing hypoxia and favoring smaller grazers over larger zooplankton, feedback widely reported for eutrophic systems (Guenther et al., 2015). These conditions, together with cyanobacterial interference, are known to disadvantage larger crustacean grazers while favoring rotifers (Ghadouani et al., 2003; Jiang et al., 2017).

### Community–environment coupling across seasons

Multivariate analyses converge on the same mechanism. Clusters and nMDS separated the samples cleanly into rainy and dry seasons (Fig. 2a, b). The dbRDA linked the dry side to salinity, temperature, pH, turbidity, TP, and silicate, grouping rotifers (*Brachionus* spp.), harpacticoids, and protozoans; the rainy side aligned with DO, Secchi, NH₄⁺, and DIN, associating *Lepadella* sp., *F. longiseta*, and *P. crassirostris* (Fig. 3). This mirrors lagoon bioindicator literature that links environmental gradients to predictable community responses (Hemraj et al., 2017). Land-use signals likely reinforce these patterns at urban margins, as shown for functional zooplankton guilds in Amazon streams (Bomfim et al., 2023).

## Conclusion

This study met its objective of identifying the seasonal drivers shaping plankton dynamics in a hypereutrophic macrotidal lagoon. The results show a clear rainy–dry seasonal partitioning, with phytoplankton primarily regulated by bottom-up factors such as dissolved inorganic phosphorus, nitrogen availability, light conditions, and flushing, while grazer pressure increased during the dry season. The multivariate models indicated that higher phytoplankton density occurred under elevated DIP and lower dissolved oxygen, together with reduced abundance of *Filinia longiseta*. Small grazers played a stronger role during the dry season, highlighting shifts between bottom-up and top-down control across the annual cycle. Although *Microcystis* peaks did not reach bloom thresholds, occasional low dissolved oxygen values reflect the system’s sensitivity to nutrient inputs and residence time. Overall, the results provide a clear framework for understanding seasonal controls on plankton structure in highly impacted estuarine lagoons.

## Data Availability

No datasets were generated or analyzed during the current study.
